# Effects of dietary quercetin on retrieved mouse oocytes and *in vitro* fertilization outcomes

**DOI:** 10.5935/1518-0557.20240073

**Published:** 2025

**Authors:** Rasrawee Chantrasiri, Pannarai Somboonchai, Waraporn Piromlertamorn, Tawiwan Pantasri, Usanee Sanmee

**Affiliations:** 1 Division of Reproductive Medicine, Department of Obstetrics and Gynecology, Faculty of Medicine, Chiang Mai University, Chiang Mai 50200, Thailand; 2 CMEx Fertility Center, Center of Medical Excellence, Chiang Mai University, Chiang Mai 50200, Thailand

**Keywords:** quercetin, oocytes, *in vitro* fertilization, embryo development

## Abstract

**Objective:**

To investigate the effects of dietary quercetin on the retrieved mouse oocytes and IVF outcomes.

**Methods:**

Female mice were divided into two groups. Mice were given 0.2 mL water without (control group) or with quercetin 30 mg/kg (quercetin group) orally via gavage, once a day for 21 consecutive days. After that female mice were superovulated for in vitro fertilization. We observed the effect of dietary quercetin on the number of retrieved oocytes, oocyte degeneration rate, fertilization rate, blastocyst formation rate, and blastocyst cell numbers.

**Results:**

There was no difference in the number of retrieved oocytes per mouse (27.3±6.7 and 27.2±5.8), oocyte fragmentation rate (28.4% and 25.0%), fertilization rate (47.4% and 50.6%) and blastocyst formation rate (34.8% and 34.7%) in the quercetin group compared to the control group. The proportion of hatching and hatched blastocyst was significantly lower in the quercetin group (17.2% and 27.8%, *p*=0.004) and significantly lower numbers of cells in TE (47.4±15.3 and 57.2±17.7) and total cells (66.2±18.5 and 77.5±20.7) compared to the control group (*p*=0.001).

**Conclusions:**

Dietary quercetin supplementation has a detrimental effect on mouse embryo quality. Moreover, it did not show any beneficial effect on the ovary in both quantity and quality. This finding raises awareness of the general use of dietary quercetin supplements in infertile females.

## INTRODUCTION

Infertility is a global health issue worldwide, found in 5-8% of developed countries and 3.5-16.7% in developing countries (Chiware *et al*., 2021). Ovarian factor is the most common causes of female infertility. This aspect of infertility is most frequently related to ovarian dysfunction, diminished ovarian reserve, or a decline in the quality of eggs with age. Although success with assisted reproductive technology (ART) treatment has increased over the last decade, many women are still suffering from unsuccessful treatment. This frequently leads to couples attempting to find other ways of increasing their chances of becoming pregnant. A method that is very popular among infertile women around the world is the taking of a dietary supplement (Kermack & Macklon, 2015).

Nowadays, there are many dietary supplements which is claimed to increase AMH levels, induce ovulation, increase fertilization rate, improve oocyte and embryo quality, and increase pregnancy rate, but the majority of these products have no proven effect (Vitagliano *et al*., 2021). In Thailand, kaffir lime juice has recently become one of the most popular products recommended for use before and during ART treatment in infertile women. Claims associated with taking this are that it increases the number of oocytes, improves the quality of oocytes, and prepares the endometrium for implantation. The active ingredient in kaffir lime juice is quercetin, 4h-1-benzopyran-4-one,2-(3,4-dihydroxyphenyl), 3,5,7 - trihydroxy-flavone. Quercetin is found in many fruits and vegetables such as berries, broccoli, apples, onions, and kaffir lime (Li *et al*., 2016; Anand David *et al*., 2016). Because of its antioxidant properties, it is used in many diseases including cancer, cardiovascular diseases, neurological diseases, obesity, diabetes, and asthma (Anand David *et al*., 2016; Kim *et al*., 1998). However, very few studies have been done on the effect of dietary quercetin on the quality and quantity of the oocytes, and the results are conflicting (Naseer *et al*., 2017; Jahan *et al*., 2018; Beazley & Nurminskaya, 2016). Moreover, no study has been carried out on its impact on in vitro fertilization (IVF) outcomes. Therefore, we decided to investigate the effects of dietary quercetin on the retrieved mouse oocytes and IVF outcomes. The objectives were to observe the number of retrieved oocytes, and investigate the fragmentation of oocytes, fertilization rate, blastocyst formation rate, and blastocyst cell numbers.

## MATERIAL AND METHODS

### Animals

Outbred female and male International Cancer Research (ICR) mice were obtained from the National Animal Institute, Mahidol University, Bangkok, Thailand. All mice were kept at the Animal Husbandry Unit, Faculty of Medicine, Chiang Mai University under controlled conditions. The room had adequate ventilation at 25±2C, under humidity of 60-70%, controlled 12-hour light/12-hour dark cycles, and fed with standard laboratory food pellet and tap water was available ad libitum. The experiments were approved by the Animal Ethics Committee (AEC) of the Faculty of Medicine, Chiang Mai University (Protocol number AF 01-009). Investigators are competent in the use and care of animals for research and are certified by the Institute of Animals for Scientific Purpose Development (IAD) and the National Research Council of Thailand (NRCT). All procedures related to mice followed the international and national guidelines for the care and use of animals for research.

### Experimental design

Quercetin (Q4951, Sigma-Aldrich, St. Louis, USA) was dissolved in dimethyl sulfoxide (DMSO; Sigma-Aldrich, St Louis, MO, USA). Based on previous studies on rats (Jahan *et al*., 2018) and rabbits (Naseer *et al*., 2017) showed a beneficial effect of dietary quercetin on ovarian follicles. Therefore, we decided to use the same dosage of 30 mg/ kg of quercetin in this study.

To assess the effect of dietary quercetin on oocytes and IVF outcomes. Sixto nine-week-old female mice were divided into two groups, control and quercetin group. Mice were given 0.2 mL water with or without quercetin orally via gavage, once a day for 21 consecutive days. The experiments were repeated six times for accurate results.

Control group: 0.2 mL water contains DMSO

Quercetin group: 0.2 mL water contains quercetin 30 mg/kg dissolve in DMSO

### Collection of cumulus - oocyte complexes (COCs)

On the 22^nd^ day of treatment, female mice were superovulated by an intraperitoneal (IP) injection of 10 IU pregnant mare serum gonadotropin (PMSG; Sigma, St. Louis, MO, USA) followed by an IP injection of 10 IU human chorionic gonadotropin (Pregnyl, Organon, Oss, The Netherlands) 48 hours later. Sixteen hours after the second injection, euthanasia was done by cervical vertebrae dislocation. The peritoneal cavity was exposed, and the two oviducts were aseptically removed and placed in Earle’s Balanced Salts Solution (EBSS; Biological Industries, Kibbutz Beit Haemek, Israel), containing 0.5% bovine serum albumin (BSA; Sigma, St Louis, MO). Cumulus-oocyte complexes (COCs) were removed from the oviduct for the insemination process. Mice that died during the treatment or did not respond to the superovulation injection were exclude from the study.

### In vitro fertilization and embryo culture

Ten to twelve-week-old male mice were sacrificed by cervical vertebrae dislocation. Both cauda epididymis were removed and placed in 1 mL of fertilization medium (G-IVF, Cook). Capacitation was allowed to proceed for 30 minutes in an atmosphere of 6% CO_2_, 5% O_2_, and 89% N_2_ at 37C. The spermatozoa were transferred to COCs drops for insemination at a final motile sperm concentration of 3.0x10^5^/mL. Control and quercetin groups were used spermatozoa obtained from the same male. After two hours, MII oocytes were transferred to a 10 L drop of cleavage medium (G1- plus; Vitrolife, Sydney, Australia) under mineral oil (Irvine Scientific). The number of oocytes per mouse and the fragmentation rate of oocytes were evaluated.

The fertilization was evaluated on the next day by counting the number of two-cell embryos. After 48 hours post-insemination, embryos were transferred to blastocyst medium (G2-plus; Vitrolife, Sydney, Australia) and cultured in similar conditions for the next 48 hours until reaching the blastocyst stage. The embryo development was evaluated every 24 hours under an inverted microscope until completion at 96 hours. Mouse blastocysts were classified as early, partial, full, expanding, hatching, and hatched blastocysts, using the criteria proposed by Gardner *et al*. (2000) for human blastocyst development.

### Differential staining of the inner cell mass (ICM) and trophectoderm cell (TE)

Differential staining was performed on all full, expanded, hatching, and hatched blastocysts. Blastocysts with intact zona were placed in 0.5% pronase (Sigma P8811) for ten minutes to lyse zona pellucida. The zona-free blastocysts were washed three times in calcium and magnesium-free phosphate buffer solution (PBS, Gibco, USA) and then exposed to rabbit anti-mouse antibody (Sigma M5774; concentration 1:1) for 30 minutes at 37C. Then washed in calcium and magnesium-free PBS and transferred into a solution containing guinea pig complement serum (Sigma S1639), 20µg/ml of propidium iodide (Sigma P4170), and 10 µg/ml of Hoechst at 37C for 15 minutes until the trophectoderm (TE) and inner cell mass (ICM) become swollen. Propidium iodide stained red in the TE nuclei of the lysing cells. The ICM nuclei were stained blue with Hoechst (Van Soom *et al*., 2001). Each blastocyst was washed and transferred on the glass slide then let air dry. The slides were mounted with glycerol and covered with a coverslip. The numbers of the TE and ICM were counted using a Nikon E600 epifluorescence microscope with an excitation filter of 515-560 nm, and a barrier filter of 590 nm, and analyzed by the LUCIA FISH program (Laboratory Imaging, Prague, Czech Republic).

### Statistical analysis

Statistical analysis was performed using SPSS program version 29. The Chi-square test was used to compare the number of oocytes, oocyte fragmentation rate, fertilization rate and blastocyst formation rates in two groups. The mean numbers of ICM, TE, total cells and ICM:TE ratio were compared by independent t-test when data distribution was normal or Mann-Whitney U test when normality could not be confirmed. A *p*-value <0.05 was considered statistically significant.

## RESULTS

A total of 56 mice were included in the study, 35 mice in the quercetin group and 21 mice in the control group. There was no difference in the number of retrieved oocytes per mouse (27.3±6.7 and 27.2±5.8), oocyte fragmentation rate (28.4% and 25.0%), fertilization rate (47.4% and 50.6%) and blastocyst formation rate (34.8% and 34.7%) in the quercetin group compared to the control group (Table 1).

**Table 1:** Effects of dietary quercetin on oocyte number, oocyte fragmentation rate, fertilization rate and blastocyst formation rate.

	Control	Quercetin	p-value
No. of mice	21	35	-
Total collected oocytes	571	954	-
No. of oocyte recovered/ mice	27.2±5.8	27.3±6.7	0.970
Oocyte fragmentation rate	143 (25.0)	271 (28.4)	0.153
Fertilization rate	289 (50.6)	452 (47.4)	0.222
Blastocyst rate	198 (34.7)	332 (34.8)	0.961

Values are presented as numbers (%).

The number of embryos that reached various stages of blastocyst development after 96 hours of culture is shown in Table 2. The proportion of hatching and hatched blastocyst was significantly lower in the quercetin group than those in the control group (17.2% and 27.8%, *p=*0.004).

**Table 2:** Mouse embryo development after 96 hours of culture.

	Control	Quercetin	p-value
No. of blastocyst	198	332	-
EB to B	58 (29.6)	107 (32.2)	0.481
FB to ExB	85 (42.9)	168 (50.6)	0.087
HB to HtB	55 (27.8)	57 (17.2)	0.004

Values are presented as numbers (%)

Chi-square test

EB, early blastocyst; B, partial blastocyst; FB, full blastocyst; ExB, expanding blastocyst; HB, hatching blastocyst; HtB, hatched blastocyst

There were significantly lower numbers of cells in TE (47.4±15.3 and 57.2±17.7) and total cells (66.2±18.5 and 77.5±20.7) of full, expanding, hatching, and hatched blastocysts in the quercetin group compared to the control group (*p=*0.001, Table 3). The numbers of cells in ICM of the blastocyst from the quercetin and the control group were comparable (18.9±5.8 and 20.3±7.7, *p=*0.121). The ICM to TE ratio was significantly lower in the control group compared to the quercetin group (0.39±0.18 and 0.42±0.14, *p=*0.014).

**Table 3:** Numbers of cells in the ICM, TE, total cell number, and the ICM to TE ratio.

	Control	Quercetin	p-value
ICM ^*^	20.3±7.7	18.9±5.8	0.121
TE ^*^	57.2±17.7	47.4±15.3	0.001
Total cell ^*^	77.5±20.7	66.2±18.5	0.001
ICM/TE ratio ^†^	0.39±0.18	0.42±0.14	0.014

Values are presented as mean±standard deviation

* t-test

† Mann-Whitney test

ICM, inner cell mass; TE, trophectoderm cell.

## DISCUSSION

The excessive production of reactive oxygen species (ROS) leads to the deterioration of the ovaries, resulting in follicular atresia, meiosis arrest, and oocyte degeneration (Immediata *et al*., 2022). To avoid these negative effects, converting ROS to water through the antioxidant pathway is necessary. Dietary antioxidant is classified as a non-enzymatic antioxidant that can eliminate ROS (Agarwal *et al*., 2005). Therefore, a significant number of infertile women are taking dietary antioxidant supplements before and during ART treatment with the expectation to increase their chance of conception (O’Reilly *et al*., 2014). We are interested in dietary quercetin because it is an active ingredient in kaffir lime juice which is widely drunk among Thai infertile women nowadays, it claims to increase the number of oocytes and enhance oocyte quality. Quercetin is a flavonoid nutrient that possesses strong antioxidant properties. It scavenges free radicals directly, inhibits lipid peroxidation to reduce the production of more free radicals and induces the expression levels of oxidative stress-related enzymes including superoxide dismutase (SOD), catalase, and glutathione peroxidase (GPX) (Baghel *et al*., 2012). Moreover, it inhibits xanthine oxidase activity to reduce superoxide formation (Lakhanpal, 2007). Quercetin has been widely used as a dietary supplement in the general population as generally regarded as safe (GRAS) and approved by the Food and Drug Administration (FDA) (Beazley & Nurminskaya, 2016). However, its utilization in the reproductive field is quite limited, its true effect on the ovary remains controversial, and the effect on IVF outcomes is still unknown.

The results of the present study indicated that dietary quercetin supplementation did not increase the number of retrieved oocytes and did not enhance the oocyte quality. The fertilization rate and blastocyst formation rate were similar to those in the non-supplemented group. However, the resulting blastocyst from mice supplemented with dietary quercetin were of lower quality as shown by the proportion of hatching and hatched blastocyst and the blastocyst cell number. However, the best indicator of blastocyst quality is the live birth rate after being transferred into the uterine horn of pseudo-pregnant mice. Blastocyst cell number, especially the number of the TE cells has also been used as the parameter to predict live birth outcome (Ahlström *et al*., 2011; Ebner *et al*., 2016). Therefore, the significantly lower number of TE cells in the quercetin-supplemented group was assumed to have a lower chance of implantation compared to the non-supplemented group.

Our results are not consistent with those from previous studies (Naseer *et al*., 2017; Jahan *et al*., 2018) that reported the advantages of dietary quercetin on the ovary, despite dietary quercetin being used in the same dose and duration. Naseer *et al*. (2017) showed an increase in the number of retrieved oocytes and good quality oocytes in rabbits that were fed with quercetin-supplemented diet evidenced by improved primordial and antral stage follicles and decreased granulosa cell apoptosis. This is consistent with the study by Jahan *et al*. (2018) which reported that rats given quercetin as a dietary supplement showed significant increases in the number of antral follicles and restoration of healthy follicles. A clear difference between their studies and our study was the use of different animal conditions. They induced condition of oxidative stress in the animals by exposing female rabbits to heat stress (Naseer *et al*., 2017) and induce polycystic ovarian syndrome (PCOS) conditions in rats (Jahan *et al*., 2018). Therefore, a possible reason for inconsistent results could be explained by the redox homeostasis mechanism. In physiologic conditions, there is a natural antioxidant system against oxidative stress counteracting the over production of ROS (Mauchart *et al*., 2023; Sugino, 2005). However, low levels of ROS are essential for cell function, to maintain the oxidative modification on many macromolecules such as transcription factors, receptors, and proteins that are essential for cell growth and controlled cell death pathway (Mauchart *et al*., 2023). Therefore, the balance between prooxidants and antioxidants within a cell is crucial for normal cell function. In stressful conditions lead to oxidative stress, providing antioxidants to reduce excess ROS that exceed the scavenging ability of natural mechanisms is undoubtedly beneficial. Nevertheless, in normal conditions without stress, super-suppression of ROS with dietary antioxidant supplement might disrupt this balance and exert pro-oxidant activity, that is, the substance induces oxidative stress and leads to poor cell function. In line with our finding that dietary quercetin supplementation in mice in normal condition reduces the embryo quality. Therefore, the use of antioxidants should be limited to conditions in which there is a specific risk of oxidative stress such as PCOS (Karuputhula *et al*., 2013) and endometriosis (Singh *et al*., 2013), two major pathologies linked to oxidative stress in women (Gupta *et al*., 2015).

In addition to the antioxidant activity, quercetin also acts as a phytoestrogen in the ovary (Yang *et al*., 2018). A high concentration of quercetin can inhibit aromatase activity and affect the biosynthesis of 17β-estradiol (Lu *et al*., 2012). Due to these activities, quercetin can negatively affect the function of the ovary. This may be another possible mechanism to explain the poor IVF outcomes in the quercetin-supplemented group in our study. Quercetin has been shown to have a dose-dependent effect on oocytes (Lu *et al*., 2012; Kang *et al*., 2013). However, it is not clear what dose of dietary quercetin supplemented is optimal to counteract the excess ROS, while maintaining a physiologic level of ROS and promoting ovarian hormone secretion for the normal development of follicles and improving the quality of oocyte. Beazley & Nurminskaya (2016) studied the effect of dietary quercetin on the natural conception of mice in the normal condition using a dose much lower than in our study, 5 mg/kg/day but a longer duration of nine months. The result showed that dietary quercetin reduced the reproductive potential of female mice by increasing birth space and decreasing the number of litters. Although this negative effect was seen only in aging mice, it is still potentially detrimental for general use. Investigation into the optimal concentration of dietary quercetin to potentially benefit general population remains an ongoing area of research.

We cannot conclude that Kaffir lime juice will give the same result as the present study. There is a difference between nature-identical antioxidants and natural-derived antioxidants (Mahmoud *et al*., 2021). Nature-identical antioxidants are pure substances that are extracted from natural sources through the process of purification and concentration (Zhu *et al*., 2023), thus they have reproducible properties including antioxidant activity. In contrast, the naturally derived antioxidants vary as regards composition and efficacy as they are usually a mixture of several substances. Flavonoids including quercetin are generally found at high concentrations in the outer layer of the kaffir lime fruits and leaves. The amount of quercetin present in juice obtained from squeezing kaffir lime fruits is questionable. The only information available is from kaffir lime leaves, that is, 100 grams of fresh leaves will contain 43±9 mg of quercetin (Butryee *et al*., 2009). Therefore, we do not know the total content of quercetin in one drink. Being a natural substance does not mean that it is not harmful. Unfortunately, the safety limits of natural antioxidants are mostly unknown (Pokorný, 2007). Fortunately, natural products are less active, it is very difficult to overdose as it requires ingesting larger quantities than a person can consume, especially in the case of pure Kaffir lime juice which has an extremely tart and bitter taste.

Based on our findings, it bears strong emphasis that regular drinking kaffir lime juice before and during ART treatment will certainly require further study into its efficacy and safety. It is also not possible to conclude that the results of this study regarding Kaffir lime juice are transferable. Careful monitoring of unanticipated the impacts of quercetin on IVF outcomes is necessary.

In conclusion, the study indicated that dietary quercetin supplementation has a detrimental effect on mouse embryo quality. Moreover, it did not show any beneficial effect on the ovary in both quantity and quality. This finding raises awareness of the general use of dietary quercetin supplements in infertile females.
